# Development and Feasibility Testing of the Clinical-Community Linkage Self-Assessment Survey for Community Organizations

**DOI:** 10.3389/fpubh.2022.797468

**Published:** 2022-05-20

**Authors:** Sarah Fishleder, Jeffrey R. Harris, Miruna Petrescu-Prahova, Marlana Kohn, Christian D. Helfrich

**Affiliations:** ^1^Health Promotion Research Center, Department of Health Systems and Population Health, School of Public Health, University of Washington, Seattle, WA, United States; ^2^US Department of Veterans Affairs Health Services Research & Development, Washington, DC, United States

**Keywords:** program evaluation (MeSH), health services, community networks (MeSH), community health services [MeSH], health promotion (source: MeSH NLM)

## Abstract

**Introduction:**

Clinical-community linkages (CCLs) can improve health, but few instruments exist to evaluate these partnerships. To address this gap, we develop and test the Clinical-Community Linkage Self-Assessment Survey (CCL Self-Assessment).

**Materials and Methods:**

We built on an existing framework and conducted a literature review to guide the design of our survey, and obtained feedback from academic, clinical, and community-based experts. To pretest the instrument, we conducted 10 think-aloud interviews with community-based health-promotion organizations. We performed feasibility testing with 38 staff from 20 community organizations, followed by criterion-validity testing.

**Results:**

The 15-item final instrument includes five domains: Nature of the Relationship, Communication, Referral Process, Feedback Loop, and Timeliness. Expert feedback included keeping the CCL Self-Assessment brief and actionable. Think-aloud interviews produced a range of revisions related to item wording, instructions, brevity, and formatting. Feasibility testing showed high response rate and ease of administration. Sites scoring high on the CCL Self-Assessment also scored high on the criterion measure.

**Discussion:**

We demonstrate feasibility, as well as face, content, construct, and criterion validity. Initial results suggest the CCL Self-Assessment survey may be used by community organizations to identify strengths and weaknesses of their linkages. Next steps include additional statistical validation and testing to determine how the CCL Self-Assessment survey works in the field as well as providing specific tools to improve linkages.

## Introduction

Clinical-community linkages (CCLs) are collaborative relationships between clinical and community organizations aimed at improving population health (1). The field of CCLs is relatively emergent (2). Both the CDC and AHRQ define a linkage as a clinic and community organization engaging in at least one of Himmelman's strategies for working together (networking, coordinating, cooperating, and collaborating). The strategies range in formality, characteristics, and degree of resource sharing. Therefore, CCLs take many diverse forms, ranging from informally sharing information, to establishing a bi-directional feedback loop with HIPAA compliant communication about each patient (1–3).

### Case Studies of CCLs

Some recent research identifies opportunities to create CCLs in diverse settings, including primary care (4), physical therapy clinics (5, 6), optometrists (7), pharmacists (8), medical teaching centers (9) and small independent practices (10). There are several reports of efforts in Hawaii focusing on a spectrum of contexts (11–13).

The literature provides case examples of CCLs that describe the various strategies and diverse settings, primarily focusing on a variety of patient outcomes. Examples include interventions focused on community health workers (14–16) system or clinical level interventions (17–21), pediatricians (22, 23), nursing students (24), and reviews describing screening and connection strategies (25). Some examples also provide descriptions of specific CCL protocols (26–28).

### Evaluation of CCL

Many of these peer-reviewed case descriptions explicitly discuss what hindered and what expanded the partnerships. These generally include either an asset or gap in: time or resources; infrastructure; and trust, rapport, or awareness of the partner (29–41). Several articles present conceptual tools within very specific contexts. These include roadmaps to address social determinants of health (42), delineating approaches to evaluate partnerships and implementation (43), and a guide on how community-based organizations can help link seniors with chronic disease management (44).

Most of the concrete evaluative tools for CCLs that do exist are in the gray literature, generally aimed at the community-based organization. These include finance and contract tools (45, 46), innovative payment models (47), implementation roadmaps (48), questionnaires, tools, and guides both in the United States (49, 50) and outside (51, 52). These resources are also written for specific contexts, are not peer reviewed, and are not based on a widely accepted CCL framework.

More research is needed to assess the effectiveness of CCLs and help organizations improve CCL quality, particularly through identification of CCL components that are key to effectiveness. Many descriptions of tools to assess CCLs do not describe validation processes, and most evaluations center on patient-level outcomes, rather than focusing on aspects of the community-clinical relationship itself, which drive the outcomes (16). A recent systematic review found the most important components affecting healthcare collaboration to be structure and processes, and standard assessment tools help focus efforts on weak components (53).

### Theoretical Framework

In order to better understand CCLs and to map existing available metrics of CCL quality, the Agency for Healthcare Research and Quality (AHRQ) created the Clinical-Community Relationships Measurement (CCRM) Framework and the CCRM Atlas (2, 3). The CCRM Framework is a comprehensive framework for evaluating CCLs that combines Etz et al.'s Bridging Model (54) with Donabedian's Structure-Process-Outcome (S-P-O) model (55)^1^. The CCRM Atlas presents this measurement framework and provides a listing of existing measures of clinical-community relationships.

The CCRM Framework identifies S-P-O *measurement domains* for each element of a linkage. Specifically, it lists seven S-P-O measurement domains related to the linkage between a clinic or clinician and a community resource. Of these seven domains, one is structural, three are related to process, and three to outcomes. However, only one (feedback & communication) has existing evaluation measures. Even these existing measures have important limitations: they are single items taken from instruments developed for different purposes, have not been validated for evaluating CCLs, and do not address all of the components defined in the domains (2, 3).

Considering the lack of peer-reviewed, validated evaluative tools that focus directly in the Clinical-Community Linkage, and to fill the gaps in evaluation measures from the CCRM Atlas, we sought to develop a pragmatic and accessible instrument that assesses a CCL's strengths and weaknesses that adhered to the conceptual model presented in the CCRM Atlas. We believe this tool will facilitate the evaluation of CCLs by community organizations and identify areas for improvement.

The purpose of this manuscript is to describe the development and testing of the Clinical-Community Linkage Self-Assessment Survey (CCL Self-Assessment), a tool for community organizations to evaluate their linkages with clinical organizations. We test the instrument for feasibility, and demonstrate face, content, construct, and criterion validity. Future development includes testing the same survey design with clinical partners to include entire CCL dyads.

## Materials and Methods

We conducted this work as part of a larger research project, Physical Therapists Recommending Enhance®Fitness to Expand Reach (PT-REFER). The project was conducted in partnership with YMCA of the USA and implemented in local YMCA Associations. A YMCA Association can consist of a single YMCA site (called branch), or be the umbrella organization for multiple branches in an area. The goal of PT-REFER was to develop and test an intervention facilitating CCLs between YMCA Associations and physical therapy clinics to increase referrals from the clinics to Enhance®Fitness, a physical activity program for older adults available through YMCA Associations (details are available at clinicaltrials.gov/ct2/show/NCT03139461) (26). The University of Washington Institutional Review Board approved this study.

We sought to develop an instrument that assesses a CCL's strengths and weaknesses and is also “pragmatic,” in terms of being: (1) actionable, (2) feasible, and (3) important to stakeholders (57). We used an established three-step method for instrument development and validation (58, 59). To ensure rigor, we developed the instrument in three sub-steps: a) we built an initial draft based on findings from a literature review and existing domains from the CCRM Framework, with its established content validity; b) we revised the draft based on feedback from academic and clinical and community-based practice experts; and c) we sought feedback from members of our intended audience—staff from community organizations that offered health promotion programs— via “think-aloud” interviews (60). We define “community-based” as organizations outside of clinical health care or public health agencies (61) and “health promotion programs” as an organized public health action that aims to enable people to increase control over, and to improve their health (62, 63). Hearing from community organization staff if and how the survey items made sense and capturing important aspects of CCLs helped establish face and construct validity. To test feasibility of the CCL Self-Assessment in practice, we administered the instrument to the 20 YMCA Associations enrolled in the PT-REFER study. We tested criterion validity by comparing the data obtained through the CCL Self-Assessment to a conceptually related but distinct survey on community outreach that also captured empirical practices related to partnership strength.

Note that we did not conduct common psychometric tests predicated on homogeneity among items, such as Cronbach's alpha and factor analysis, which are only appropriate under assumptions of a shared cause (64, 65). The CCL Self-Assessment is a formative measure, in which the domains collectively “form” the latent construct. These types of models have neither internal consistency, nor the same antecedents and consequences as reflective measures. Homogeneity of items is not important for cause indicators; however, the specific items included is important for cause indicators because the latent variable is caused by them, and therefore omitting an item may invalidate the indicator. Criterion validation is one of the few objective ways of evaluating cause indicators. Therefore, tests of homogeneity are at best irrelevant and at worst can produce spurious findings (65, 66).

### First Step: Instrument Development

#### The CCRM Domains and Content Validity

Structure and process are considered the most actionable components of Donabedian's S-P-O model (67). Therefore, we focused our instrument on measuring the structure and process domains of CCLs, as described in the CCRM Framework, and excluded the outcome domains. *Content validity* refers to how well items taken together constitute an adequate operational definition of the construct, and the degree to which the items are relevant, representative, and comprehensive of the construct (68). Content validity was established in the rigorous development of the CCRM Framework domains, by identifying items through literature review and using domains from the CCRM Framework, followed by iterative feedback on the instrument from academic and clinical and community-based practice experts and members of our intended audience. We built the foundation of our conceptual model on these established, peer-reviewed, and widely accepted materials.

The structure and process domains, as they appear in the CCRM Framework (2, 3), are: (1) nature and strength of inter-organizational relationship, (2) feedback and communication, (3) referral process, and (4) timeliness. We present the definitions and how each of our final items align to them below (see Section Obtaining Expert Feedback).

#### Identifying Existing Instruments

**Figure 1:**
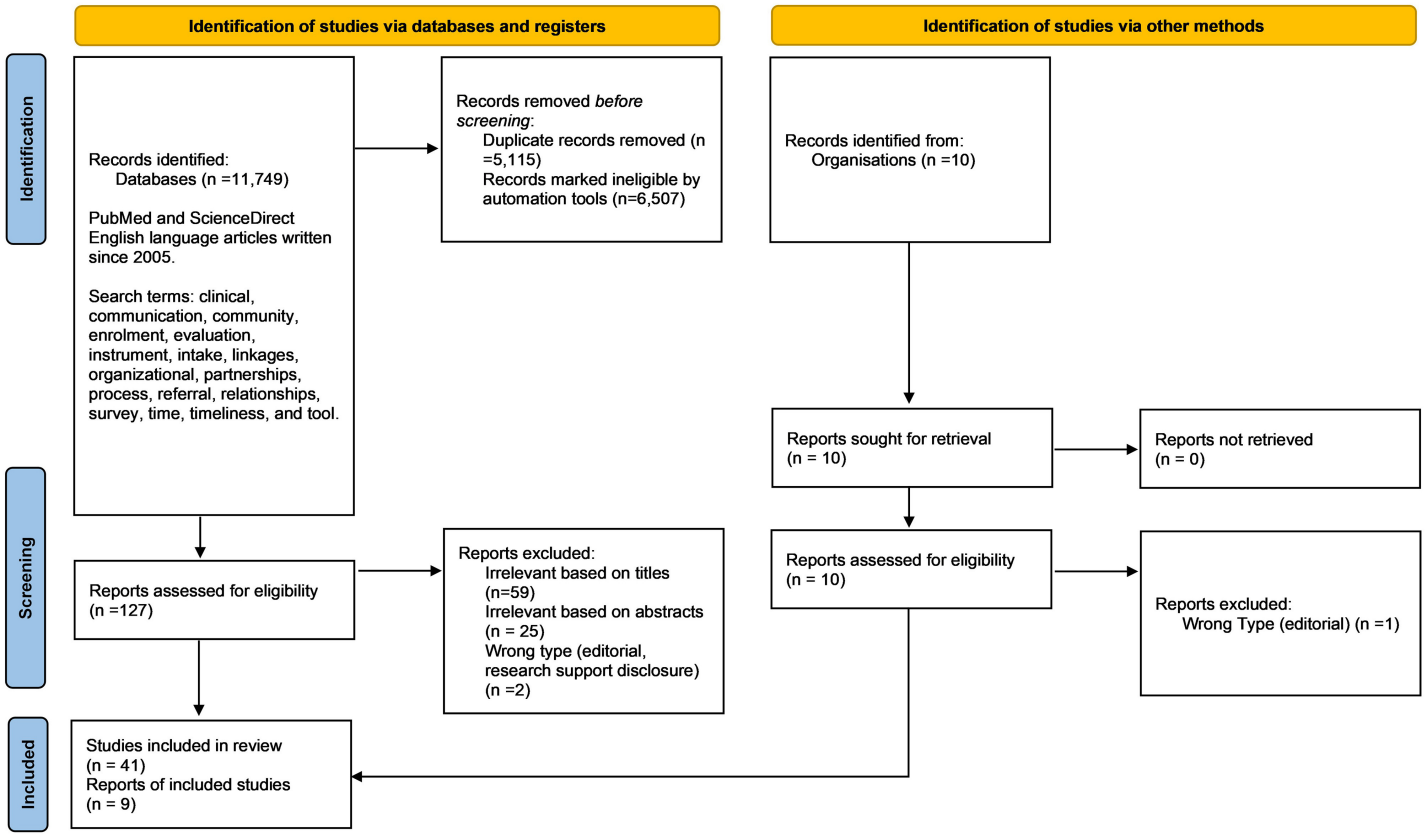
PRISMA flow diagram for the systematic review detailing the search process, number of sources identified, screened, and included.

To explore whether published CCL instruments existed for these domains, we conducted a two-part comprehensive literature review, searching PubMed and ScienceDirect for English language articles written since 2005. We began by searching for real-world example of formal CCLs, including reports, examples, applications, and evaluations. We moved on to a literature review related to the CCRM Framework domains listed above. Search terms included: *clinical, communication, community, enrollment, evaluation, instrument, intake, linkages, organizational, partnerships, process, referral, relationships, survey, time, timeliness*, and *tool*. A diagram of the process is depicted in Figure 1, and a summary of our citations are depicted in Table 1.

**Table 1:** Summary of publications included literature review.

**Citation**	**Partners or settings**	**Program or population of focus**	**Purpose with link to resource (if applicable**	**Number of people or entities**	**Region**
(4)	Primary care providers	Adults at risk for cardiovascular disease (CVD)	Assess primary care providers' awareness of and referral to physical activity-related behavioral counseling services	1256 primary care practitioners	United States
(5)	Physical therapy clinics	Older adults at risk for falls	Describe knowledge and characteristics of physical therapists and physical therapist assistants	444 physical therapists	United States
(6)	Physical therapy clinics	Older adults	Assesses capacity of physical therapists to participate in CCLs with physical activity programs	30 physical therapists	United States
(7)	Optometrists	Older adults	Examines the potential for optometrists' referrals to exercise programs	42 optometry patients 268 optometrists	Texas
(8)	Pharmacists	General population	Assess community pharmacists' involvement and interest in CCLs	500 pharmacists	Ohio
(9)	Medical teaching centers	Persons facing food insecurity	Describes symbiotic relationship between nursing school and the regional food bank	n/a	Virginia
(10)	Small independent clinical practices, community health workers	Low-income, underserved patients	Describes practice facilitation to support community health worker integration in small, independently owned practices.	n/a	United States
(11)	Community Health Workers, Health Systems-Based Programs, Community Health Center-Based Programs, Provider-Based Programs, Queens Health Care System	General population	Introductory article in an issues dedicated to describing innovations in community-clinical linkages in various contexts across Hawai‘i.	n/a	Hawaii
(12)	Community health workers	Healthcare staff, community health staff	Describes educational programs as a solution to improve trust and rapport between health care and community health workers	n/a	Hawaii
(13)	Health care clinic, electronic health records	Clinic population	Describes integration of social determinants data into clinical care via electronic health records.	3 local health care delivery systems	Hawaii
(14)	Community health workers	Latino/a populations	Describes a CCL intervention to improve emotional well-being led by community health workers	189 participants	US-Mexico border
(15)	Community health workers	Underserved populations	Describes workforce development model to effectively connect communities with care	n/a	Maryland
(16)	Community health workers	Community health worker interventions using CCL models	Presents a scoping literature review, synthesizing evidence of community health workers in creating and sustaining CCLs aimed at improving individual health outcomes.	n/a	United States
(17)	Health care providers, Maternal Infant Health Program (MIHP)	Medicaid-Eligible Pregnant Women	Assesses operationalization of CCL linkage strategies effectiveness to improve MIHP participation and other service use.	2 practice sites	Michigan
(18)	Public health agencies, clinical and community settings	Patients at risk for hypertension	Presents examples of agencies applying a framework after a learning collaborative	31 state and territorial public health agencies	United States
(19)	Data systems, clinical and community settings	The Childhood Obesity Data Initiative (CODI)	Describes a participatory framework to enhance and implement changes in an existing distributed health data network (DHDN) infrastructure to support linkages across sectors and systems.	3 health care systems, 2 community partners	Denver, Colorado
(20)	Pediatric practices	Children at high risk for obesity	Describes the creation of an online interactive community resources map	11 parents, 5 community partners, 2 pediatricians 3 obesity-built environment experts	Eastern Massachusetts
(21)	The American Medical Association (AMA), YMCA of the USA	Medicare patients with pre-diabetes	Describes implementation, and evaluation of quality improvement strategies to increase routine screening, testing, and referral to diabetes prevention programs (DPPs)	26 primary care practices and health systems	United States
(22)	Pediatric practices	Children in poverty	Narrative review of childhood poverty programs that use specific methods: co-design, community organizing, and community-engaged quality improvement	n/a	United States
(23)	Pediatric practices	Keeping Infants Nourished and Developing (KIND)	Describes the design, implementation, refinement, and evaluation of a collaborative intervention focused on food-insecure families with infants.	1,042 families with infants	Cincinnati, Ohio
(24)	Academic-community partnership, Nursing students	Older adults, low-income housing communities	Describes partnership to develop high-impact community-based learning experiences to support personal health goal attainment.	n/a	North Carolina
(25)	Health care entities	Patients with food insecurity	Landscape assessment describing strategies for screening patients and connecting them to food resources	n/a	United States
(26)	Physical therapy clinics, YMCAs	Older adults, Physical Therapists Recommending Enhance®Fitness to Expand Reach (PT-REFER)	Describes protocol to test an intervention focused on developing CCLs to increase referrals from physical therapy clinics to an evidence-based group exercise program	20 YMCA associations	United States
(27)	Primary care practices, Community health workers, electronic health records	South Asians with uncontrolled diabetes	Describes the protocol for a multi-level, CCL intervention to improve glycemic control using electronic health records and community health workers	886 individuals, 20 primary care practices	New York City
(28)	Community health workers, electronic health records	Latinos with chronic diseases, Linking Individual Needs to Community and Clinical Services (LINKS)	Presents protocol for community health worker-led CCLs to reduce chronic disease risk and promote emotional well-being through utilization of electronic health records	Estimated 250 participants	U.S.-Mexico border
(29)	Community organizations	Diabetes self-management education (DSME)	Document the landscape of DSME services in the state, focusing specifically on challenges and recommendations	17 interviewees	Hawaii
(30)	Clinical and community settings	Patients with hypertension	Examines partnerships for blood pressure control, their facilitators and barriers, and ways public health departments can foster partnerships.	41 staff members	Washington State
(31)	Physical therapy clinics, YMCAs	Older adults, Enhance®Fitness	Tested a capacity-building intervention that included a structured toolkit and technical-assistance calls intended to increase referrals to programs offered at YMCAs	20 YMCA associations	United States
(32)	Academic-VA community clinical research partnerships	Veterans	Authors reflect on the challenges and rewards of implementing partnerships with the aim of assisting new VA investigators and VA collaborators.	n/a	California
(33)	Primary care practices	Patients with pre-diabetes, clinical-community linkages to prevent diabetes (CC-Link) study	Describes the development and implementation of an integrated framework to guide clinic-community linkages	10 primary care practices	Indiana
(34)	Faith-community nurses, community organizations	Faith community health partnership	Case study reporting factors leading to the sustainability of a specific CCL	18 individuals	California
(35)	YMCA of the USA	General population	Presents practices based on the experience of local YMCAs and YMCA of the USA in establishing clinic-to-community partnerships throughout the country that can influence clinical cost and quality measures.	n/a	United States
(36)	Evidence based programs	Prevention and wellness trust fund initiative	Social network analysis perspective to explore (a) the range of contributions made by CCL network members to support the delivery of preventive services and (b) influences on the ability of these partnerships to sustain service delivery	Social networks held within each of 9 partnerships	Massachusetts
(37)	Federally qualified health centers	Diabetes prevention and hypertension management	Case study to understand how FQHCs engaged community health workers, the types of CCLs the community health workers promoted, and the facilitators of and barriers to those linkages	6 administrators/clinicians, 7 community health workers	Hawaii
(38)	Cancer prevention and control research network (CPCRN), clinical and community settings	HPV vaccination	Describes evaluation of HPV-related CCLs (CCLs) to understand their components, processes, and outcomes to increase HPV vaccination.	9 CCLs	United States
(39)	Federally qualified health centers, community organizations	Hypertension patients, underserved populations	Process evaluation of a case study where a community-based organization acted as an external facilitator, and employed a collaborative partnership model to catalyze implementation of evidence-based interventions in safety net settings.	3 federally qualified health centers	Los Angeles, California
(40)	Health care providers	Healthcare systems	Synthesized expert views about how healthcare systems transform and partner to improve population health. Creates and illustrates a proposed model.	9 organizations	United States
(41)	Clinical and community settings	Cancer patients	Describes reach, partnerships, products, benefits, and lessons learned from the community-based participatory research to reduce cancer health disparities	25 community network programs	United States
(42)	Primary care practices, community organizations	Children, primary care clinical practices	Presents a roadmap to help structure primary care approaches to social determinants through the development of comprehensive and effective collaborations between the primary care setting and community partners	n/a	United States
(43)	Clinical and community settings	Prevention and wellness trust fund (PWTF)	Methods paper describing approach for evaluation of implementation of evidence-based prevention interventions by PWTF partnerships	9 leadership interviews, 172 staff survey, 72 social network analysis, 24 staff interviews	Massachusetts
(44)	Primary care practices, community organizations	General population	Guide on how community-based organizations can help link seniors with chronic disease management	n/a	United States
(45)	Aging and disability community-based organizations	Older adults and people with disabilities	Group of assessment tools designed to guide organizations through the process of successfully preparing, securing, and maintaining contracts with the health care sector	n/a	United States
(46)	Community-based organizations and their health system partners	High-need, high-cost patients	Return on Investment Calculator Tool designed to help plan sustainable financial arrangements to fund the delivery of social services	n/a	United States
(47)	National meals on wheels	Older adults, homebound seniors	Report describing Meals on Wheels American's Medicare Advantage (MA) plan New service provides a home-delivered meal benefit in conjunction with other supportive services, and is financed through a Pay for Success (PFS) transaction	n/a	United States
(48)	Health leads	General population	Essential Needs Roadmap Provides a group of resources providing guidance on implementing or scaling social health initiatives, including a section on community partnerships	n/a	United States
(50)	Center for healthcare strategies	Low-income and/or vulnerable populations.	Partnership Assessment Tool for Health (PATH) Tool created to help partnering organizations work together more effectively to maximize the impact of the partnership.	n/a	United States
(49)	National Counsel on Aging	Older adults	NCOA Partnership Assessment Tool Tool provides a method for assessing key areas of your network, including: partnership alignment, organization culture integration, network construction (infrastructure), defining responsibilities	n/a	United States
(51)	Safer care Victoria (government organization)	General population	Partnering in healthcare framework and self-assessment tool Tools developed to support practical strategies and partnerships between consumers and health services	n/a	Australia
(52)	Center for the advancement of collaborative strategies in health	General population	Partnership Self-Assessment Tool Questionnaire that partners can complete to examine the strengths and weakness of the partnership	n/a	Canada
(53)	Health sector entities	Disaster victims	Systematic review aimed to identify the components affecting collaboration of health sector in disasters	n/a	Worldwide

The literature review identified few papers, none of which included information that could be used in the development of the instrument. Most papers we identified addressed organizational collaboration in unrelated contexts; focused on measuring patient-level outcomes; or dealt with case studies of CCLs, new methods of adding features to CCLs, and various strategies for improving CCLs.

We also reviewed three potentially relevant survey instruments referenced in the CCRM Atlas (69–71). Of these, only the Continuity of Care Practices Survey – Practice Level [CCPS-P] (71) included items related to our domains of interest. Because the instrument had been developed in the substance abuse context, we adapted six items from this instrument to reflect a broader referral process. We edited the text, and adjusted item order to follow the logical progression of the referral process. The adapted items are questions 3a, 3b, and 3c of the final CCL Self-Assessment (see Table 2: CCL Self-Assessment).

**Table 2:** Complete clinical-community linkage self-assessment survey.

**The following statements characterize how your organization interacts with clinical partners. Please rate which response most accurately represents each statement**.		**We do not do this (0)**	**We do this for less than half our partners** **(1)**	**We do this for more than half our partners (2)**	**We do this for nearly all our partners** **(3)**
**1. Nature of the relationship**					
**1.a**	Our organization and our clinical partners have a system in place to share information (electronic or otherwise). *(e.g., We exchange information about how we each support patients.)*	0	1	2	3
**1.b**	Our organization and our clinical partners change activities to make accessing each other's services or resources easier for patients. *(e.g., We change our service schedules so patients can visit our organization directly after their clinic visit.)*	0	1	2	3
**1.c**	Our organization and our clinical partners share resources to better connect patients with clinics and with our programs. *(e.g., We share the costs of employing a part-time marketing and outreach coordinator.)*	0	1	2	3
**1.d**	Our organization and our clinical partners enhance each other's capacity to support patients. *(e.g., We provide skill-development training for each other's staff.)*	0	1	2	3
**1.e**	Our organization and our clinical partners facilitate insurance reimbursement for patient participation in our programs. *(e.g., We advocate together to help get our programs covered by the patient's health insurance.)*	0	1	2	3
**2. Communication**					
**2a**	Our organization and our partners have each designated specific people we can contact when needed.	0	1	2	3
**2b**	Our organization has formal or informal procedures that enable consistent connection with our clinical partners, either on the phone, by email, fax, text, in writing, or in person.	0	1	2	3
**3. Referral process**					
**3a**	Our clinical partners notify our organization when they refer a patient, rather than giving the referral only to the patient.	0	1	2	3
**3b**	Our clinical partners set up an appointment for referred patients with a member of our staff.	0	1	2	3
**3c**	Our clinical partners review the patient's discharge summary or discuss health concerns with our organization prior to a patient's first session.	0	1	2	3
**3d**	Our organization has the capacity to assess referral rates *(i.e., how many patients are being sent)* from our clinical partners.	0	1	2	3
**4. Feedback loop**					
**4a**	After receiving a referral, our organization confirms with our clinical partners that the referred patient is attending the program.	0	1	2	3
**4b**	After receiving a referral, our organization receives specific information from the clinical partners about a patient (e.g., if their condition changes).	0	1	2	3
**4c**	After a referred patient enrolls, our organization regularly sends our clinical partners information about patients' outcomes *(e.g., once every program cycle)*.	0	1	2	3
**5. Timeliness**					
**5a**	After receiving a referral from our clinical partners, patients are able to enroll in our program within the shortest possible amount of time *(e.g., less than two weeks)*.	0	1	2	3

*Sum your score across each domain. Domains with lower scores indicate weaker linkages areas. Domains with higher scores indicate stronger linkage areas*.

#### Generating New Items and Designing the Instrument

We developed an initial draft of the CCL Self-Assessment, which contained 29 draft items representing core concepts based on the definition of each of the CCRM Framework domains of interest. The draft items included an initial draft of the six items from the Continuity of Care Practices Survey – Practice Level mentioned above. Each item in our initial draft was a statement (e.g., *The partnership with our clinical partner requires extensive sharing of responsibilities*.), with a 7-point response scale, indicating to what extent statements applied to a single partnership, and an option “Not able to answer.” A score of 1 indicated “To little or no extent” and a score of 7 indicated “To a very great extent.” The draft instrument contained the following: *Nature of the relationship (*6 items); *Referral process* (7 items); *Feedback and Communication* (6 items); *Timeliness* (2 items).

#### Obtaining Expert Feedback

We solicited feedback on the draft instrument through open, in-person discussion from three expert panels affiliated with the PT-REFER study. Each discussion was led by an experienced facilitator while a second person took notes. The three panels included: project co-investigators not involved in instrument development (9 members), the Project Advisory Group (10 members), and the Scientific Advisory Board (5 members). Panel-member expertise included: research and practice (in the fields of physical therapy, psychiatry, public health, and communication); community organization operations (such as exercise, health, and evidence-based programs; and payer engagement strategies); dissemination and implementation science; organizational behavior and behavioral science; clinical practice and management; evaluation; statistics; and CCLs. We incorporated feedback after each panel review. The panels noted that the CCL Self-Assessment should be both useful and actionable, and suggested adding a component to guide improvement (i.e., a toolkit or implementation guide). We also received feedback related to the instrument's format, scoring, clarity, brevity, content, and flow.

We initially focused the instrument on a single partnership to enable community organizations to choose which partnership to evaluate. However, the experts on our panels advised that examining the partnerships globally would identify variability among partnerships, broad areas needing improvement, and reduce the danger of selection bias (from organizations only evaluating their weakest or strongest partnerships). Further, this would reduce burden, make instrument simpler, improve the likelihood of the CCL Self-assessment being used, and be more useful in general.

As a result of panel feedback, we reframed the instructions and items clarified that the survey focused on how the organization interacts with all their clinical partners, rather than evaluating a single clinical partner. We adjusted the formatting and wording to make items clearer and shorter, and to make the survey appear cleaner (i.e., making the document more accessible and inviting; formatted to facilitate consumption of information with less effort). We split the Feedback and Communication domain into two categories to reflect the logical flow and progression of referral activities. In addition, we identified items with a large degree of overlap, where we could eliminate items without compromising content validity, and shortened the instrument to a total of 15 items.

We also adjusted the survey-response scale to represent how consistently the action is taken with partners because it was more intuitive for respondents in thinking about their CCLs. The final draft moved from a seven-point Likert scale to a forced choice Likert scale with 4 options: (1) We do not do this; (2) We do this for less than half our partners, (3) We do this for more than half our partners, (4) We do this for nearly all our partners.

#### Final Items and Definitions

The final order of item categories was: nature of the relationship, communication, referral process, feedback, and timeliness. Every item in our instrument drew directly from these definitions of the domains. We present the definitions below, and the final items drawn from each. The final instrument is presented in Table 2: CCL Self-Assessment.

##### Structure Domain

**Nature and strength of inter-organizational relationship** was defined as: the level of intensity of a relationship between a clinic/clinician and community resource, based on Himmelman's (2002) definitions of networking, coordinating, cooperating, and collaborating. This includes the degree to which the relationship can overcome common barriers of working together— time, trust, and turf (72). Specifically, Himmelman defines:

*Networking* as exchanging information for mutual benefit.

*Coordinating* as exchanging information and altering activities for mutual benefit and to achieve a common purpose.

*Cooperating* as exchanging information, altering activities, and sharing resources for mutual benefit and to achieve a common purpose.

*Collaborating* as exchanging information, altering activities, sharing resources, and enhancing the capacity of another for mutual benefit and to achieve a common purpose.

The five final **Nature of the Relationship** in the CCL Self-Assessment items were:

a. Our organization and our clinical partners have a system in place to share information (electronic or otherwise). (e.g., We exchange information about how we each support patients.)
b. Our organization and our clinical partners change activities to make accessing each other's services or resources easier for patients. (e.g., We change our service schedules so patients can visit our organization directly after their clinic visit.)
c. Our organization and our clinical partners share resources to better connect patients with clinics and with our programs. (e.g., We share the costs of employing a part-time marketing and outreach coordinator.)
d. Our organization and our clinical partners enhance each other's capacity to support patients. (e.g., We provide skill-development training for each other's staff.)
e. Our organization and our clinical partners facilitate insurance reimbursement for patient participation in our programs. (e.g., We advocate together to help get our programs covered by the patient's health insurance.)

##### Process Domains

**Feedback and communication** was defined as: the level and means of communication between the community resource and the clinic/clinician (72).

The two final **Communication** items in the CCL Self-Assessment were:

a. Our organization and our partners have each designated specific people we can contact when needed.
b. Our organization has formal or informal procedures that enable consistent connection with our clinical partners, either on the phone, by email, fax, text, in writing, or in person.

The three final **Feedback Loop** items in the CCL Self-Assessment were:

a. After receiving a referral, our organization confirms with our clinical partners that the referred patient is attending the program.
b. After receiving a referral, our organization receives specific information from the clinical partners about a patient (e.g., if their condition changes).
c. After a referred patient enrolls, our organization regularly sends our clinical partners information about patients' outcomes (e.g., once every program cycle).

**Referral process** was defined in the CCRM Atlas as: data (e.g., frequency) related to the process of developing, obtaining, and confirming a referral (72).

The four final **Referral process** items in the CCL Self-Assessment were:

a. Our clinical partners notify our organization when they refer a patient, rather than giving the referral only to the patient.
b. Our clinical partners set up an appointment for referred patients with a member of our staff.
c. Our clinical partners review the patient's discharge summary or discuss health concerns with our organization prior to a patient's first session.
d. Our organization has the capacity to assess referral rates (i.e., how many patients are being sent) from our clinical partners.

**Timeliness** was defined in the CCRM Atlas as: the amount of time it takes for clinical preventive services to be delivered when clinicians make referrals to community resources (72).

The one final **Timeliness** items in the CCL Self-Assessment were:

a. After receiving a referral from our clinical partners, patients are able to enroll in our program within the shortest possible amount of time (e.g., less than 2 weeks).

#### Think-Aloud Interviews, Face Validity, and Construct Validity

To gain insight into the instrument's usability, we performed *think-aloud interviews* with the intended audience—community organization staff. This type of cognitive pre-testing reveals how users understand and navigate a survey and its questions, and identifies problems with wording or comprehension (73). It assesses *face validity*, the extent to which a test subjectively appears to measure its intended aims (i.e., the relevance of a test as it appears to test participants) (59). It also captures *construct validity*, the appropriateness of inferences made on the basis of tested items, specifically whether a test measures the intended construct (74, 75). To ensure we were building a pragmatic tool (57), we asked participants about the instrument's relevance to their jobs, the burden to complete, whether they would use it, and in what context, and how it could be more useful.

#### Think-Aloud Interview Procedures

We recruited interview participants using purposive sampling to gain the most information possible with efficient use of resources (76). We purposively sampled from (a) community organizations that offered health promotion programs; and (b) had received clinical referrals previously. Further, we were confident in our ability to engage with the interview participants as they had previously participated in related research. We sent an informational flier to community organizations that were in the greater Seattle area. We had no specific requirements about participant position in the organization (i.e., management vs. staff) because we were designing the survey to be completed by anyone at the organization. All organizations offered health-promotion programs (e.g., aging services, tobacco cessation, or breastfeeding support) and received clinical referrals. Interested participants responded via email.

We conducted each interview at the participant's office, with one researcher taking field notes and a second conducting the audio-recorded interviews. Interviews lasted 40–75 min, and participants received a $75 incentive (determined by the fair value and time commitment estimates in the Seattle areas, as determined by the University of Washington). In accordance with accepted think-aloud interview procedures, participants narrated their thoughts as they reviewed the entire printed CCL Self-Assessment. We probed as little as possible to avoid influencing responses but reminded participants to think aloud (73). After each page, we asked participants to explain questions in their own words. We asked explicitly about every item and prompt, heading, response option, and scale to determine if they were appropriate and intuitive, and whether anything should be reworded or re-ordered. At the end, we debriefed overall impressions, usefulness, and length, and asked about the context in which participants might use the instrument. We revised the instrument throughout the cognitive testing period.

### Second Step: Feasibility Testing

Feasibility studies help determine if a proposed piece of research (e.g., the CCL Self-Assessment) can realistically be completed (77). We tested the survey (Table 2) for feasibility with staff of the 20 YMCA Associations already enrolled in the PT-REFER study. We administered the survey online using REDCap electronic data-capture tools (REDCap, RRID:SCR_003445) hosted at the University of Washington (78, 79) through grant support (UL1 TR002319, KL2 TR002317, and TL1 TR002318 from NCATS/NIH).

We sent an initial invitation via email to our primary contact at each of the 20 YMCA Associations and followed up with reminders. We received a response from every YMCA primary contact (*n* = 20). At the end of the survey, we asked the primary contact to provide the name and email address of a second person who worked at their YMCA Association and was knowledgeable about current clinical partnerships. Eighteen of the primary contacts were able to provide a second contact, to whom we sent the same survey email invitation and reminders (*n* = 18). This allowed us to increase the sample size and include multiple perspectives.

#### Scoring

In the final instrument (Table 2), each item was rated on a categorical scale from zero to three. The points assigned for each response option were: We do not do this = 0; We do this for less than half our partners = 1; We do this for more than half our partners = 2; We do this for nearly all our partners = 3. We summed item scores for each domain, and the final survey was summed across all five domains. The total survey had a score range from 0 to 45 points. Numbers of items and possible points, by domain, were: Nature of the Relationship (5 items, 15 points); Communication (2 questions, 6 points); Referral Process (4 items, 12 points); Feedback Loop (3 items, 9 points); and Timeliness (1 item, 3 points).

The respondent would sum their score across each domain. Domains with lower scores indicate linkages areas that could be strengthened. Domains with higher scores indicate linkage areas are already strong.

### Third Step: Criterion Validation

#### Criterion Measure: Outreach Practices Survey

Criterion validity refers to how well operationalized scores correlate with a representation of non-test criteria (i.e., the criterion measure) (80). For the criterion measure, we calculated a summary score from sections of a conceptually related but distinct PT-REFER instrument, the Outreach Practices Survey. This instrument captures self-reported partnership activities and empirically measures practices of partnerships, among other concepts. Although it captures comparable activities, it is not a self-assessment of overall partnership strength and was not developed in collaboration with partner organizations, and therefore does not fill the same needs as the CCL Self-Assessment.

Scores were based on seven items: the number of clinical partners (3 categorical options), frequency of communication (6 categorical options), clinical partner's response to the organization's outreach efforts (6 categorical options), strategies used to maintain relationships (checklist of 7 items), level of support from senior leadership (6 categorical options), strategies used to facilitate outreach (checklist of 6 items), and major barriers (checklist of 6 items). Categorical options were assigned points from zero to 3, or zero to 6. Checklist items were assigned one point for each response; except barriers, which were assigned one negative point per response. Scores were summed across all items, such that higher scores indicated stronger connections. The mean score of this survey was 13.4, with a standard deviation of 6.0. High scores on the CCL Self-Assessment were expected to align with high scores on the Outreach Practices Survey.

#### Criterion Validation Analysis

We administered the CCL Self-Assessment online with 38 YMCA representatives in the PT-REFER study using REDCap electronic data-capture tools hosted at the University of Washington (REDCap, RRID:SCR_003445) (78). Initial invitations were sent via email, and followed up with 3 reminders sent every 4 days. We compared the summary scores from the Outreach Practices Survey to the scores from the CCL Self-Assessment in STATA (Stata, RRID:SCR_012763) version #13.1 using simple linear regression and Pearson's correlation coefficient.

As a secondary descriptive analysis, we tested each of the five CCL Self-Assessment domains against the summary score of the Outreach Practices Survey using the same analytical techniques. Because of the exploratory nature of the secondary analysis and the small number of repeated tests (*n* = 5), we did not adjust for multiple measures.

## Results

### First Step: Instrument Development

#### Think Aloud Interviews

Interviewees found the CCL Self-Assessment accessible, low-burden, and useful. Feedback included suggestions for a) rewording an item, scale, or set of instructions for clarity or precision; b) cutting words for brevity, accessibility, and reduction of visual clutter; and c) formatting to emphasize important points. By the 10th interview, no new insights were emerging, so no additional interviews were conducted.

All participants found the survey length to be appropriate. They suggested retaining all 15 items and offering the survey in both paper and electronic formats. No respondent suggested focusing the survey on single partnerships. All said the survey would be useful to evaluate the strength of their linkages and that they could see themselves using the survey. Respondents were also asked explicitly about each item, the set of prompts, if the scale seemed sensible, and if the answer choices fit. They were asked if the section headers made sense and were helpful.

In order to make the instrument more useful and actionable, participants wanted a separate toolkit with specific action steps that mapped to the instrument. Several participants noted the CCL Self-Assessment would be most useful and applicable to a program manager, rather than to staff working directly with program participants.

### Second Step: Feasibility Testing

Thirty-eight staff from 20 YMCA Associations completed the CCL Self-Assessment online; two YMCA Associations had only one staff member participate. The sample was geographically diverse, representing 13 states across the U.S. Of the 38 participants, 34 (89.5%) were females. Five (13%) YMCA Association staff members reported having no clinical partnerships, 14 (37%) reported having 1–5 clinical partnerships, and half (50%) reported at least 6 clinical partnerships. Half of the YMCA Associations consisted of a single branch, while the other half consisted of multiple branches.

Summary data for the CCL Self-Assessment, its domains and specific items are presented in Table 3. All individual item scores predicted whether the corresponding domain score was below or above the midpoint of its range. Put differently, if the domain score was above the midpoint, all corresponding items were also above the midpoint. On all but two of the items, this small sample used the full scoring range (0–3). Standard deviations were generally similar across items, fitting the normal distribution. Timeliness scored highest on the CCL Self-Assessment, while Referral Process scored lowest.

**Table 3:** Descriptive data and results of criterion validity testing of the clinical-community linkage self-assessment, 2017 (*n* = 38)^*a*^.

	**Descriptive data**				**Simple linear regression**				**Pearson's correlation**	
	**Mean**	**Median**	**SD**	**Range**	**β**	**p**	**CI**		**Coef**.	**p**
**Primary analysis**										
**Survey total (45 points possible)**	**15.1**	**15.5**	**8.1**	**0–36**	**0.89**	**0.001**	**0.44**	**1.34**	**0.71**	**<0.001**
**Secondary analysis by domain**										
**Nature of the Relationship (15 points possible)**	**4.2**	**3.5**	**3.3**	**0–10**	**0.31**	**0.001**	**0.14**	**0.48**	**0.50**	**0.001**
Our organization and our clinical partners have a system in place to share information (electronic or otherwise).	1.4	1.0	1.1	0**–**3						
… change activities to make accessing each other's services or resources easier for patients.	0.8	0.0	1.0	0**–**3						
… share resources to better connect patients with clinics and with our programs.	0.9	1.0	1.1	0**–**3						
… enhance each other's capacity to support patients	0.9	1.0	1.0	0**–**3						
… facilitate insurance reimbursement for patient participation in our programs.	0.2	0.0	0.5	0**–**2						
**Communication (6 points possible)**	**3.7**	**4**	**1.8**	**0–6**	**0.17**	**<0.001**	**0.06**	**0.30**	**0.72**	**<0.001**
Our organization and our partners have each designated specific people we can contact when needed.	1.8	2.0	1.0	0**–**3						
Our organization has formal or informal procedures that enable consistent connection with our clinical partners, either on the phone, by email, fax, text, in writing, or in person.	1.9	2.0	1.0	0**–**3						
**Referral Process (12 points possible)**	**2.8**	**2**	**2.4**	**0–9**	**0.19**	**0.001**	**0.09**	**0.30**	**0.60**	**<0.001**
Our clinical partners notify our organization when they refer a patient, rather than giving the referral only to the patient.	0.9	1.0	1.0	0**–**3						
…set up an appointment for referred patients with a member of our staff.	0.4	0.0	0.8	0**–**3						
…review the patient's discharge summary or discuss health concerns with our organization prior to a patient's first session.	0.5	0.0	0.6	0**–**2						
Our organization has the capacity to assess referral rates *(i.e., how many patients are being sent)* from our clinical partners.	1.1	1.0	1.1	0**–**3						
**Feedback Loop (9 points possible)**	**2.4**	**2.5**	**2.3**	**0–9**	**0.15**	**0.049**	**0.00**	**0.30**	**0.47**	**0.03**
After receiving a referral, our organization confirms with our clinical partners that the referred patient is attending the program.	1.0	1.0	1.0	0**–**3						
… our organization receives specific information from the clinical partners about a patient (e.g., if their condition changes).	0.5	0.0	0.7	0**–**3						
After a referred patient enrolls, our organization regularly sends our clinical partners information about patients' outcomes (e.g., once every program cycle).	0.9	1.0	0.9	0**–**3						
**Timeliness (3 points possible)**	**1.9**	**2**	**1.0**	**0–3**	**0.06**	**0.23**	**−0.38**	**0.15**	**0.35**	**0.13**
After receiving a referral from our clinical partners, patients are able to enroll in our program within the shortest possible amount of time (e.g., less than two weeks).	1.9	2.0	1.0	0**–**3						

a *The regression and correlation examine the CCL Self-Assessment and the Outreach Practices Survey*.

### Third Step: Criterion Validation

**Figure 2:**
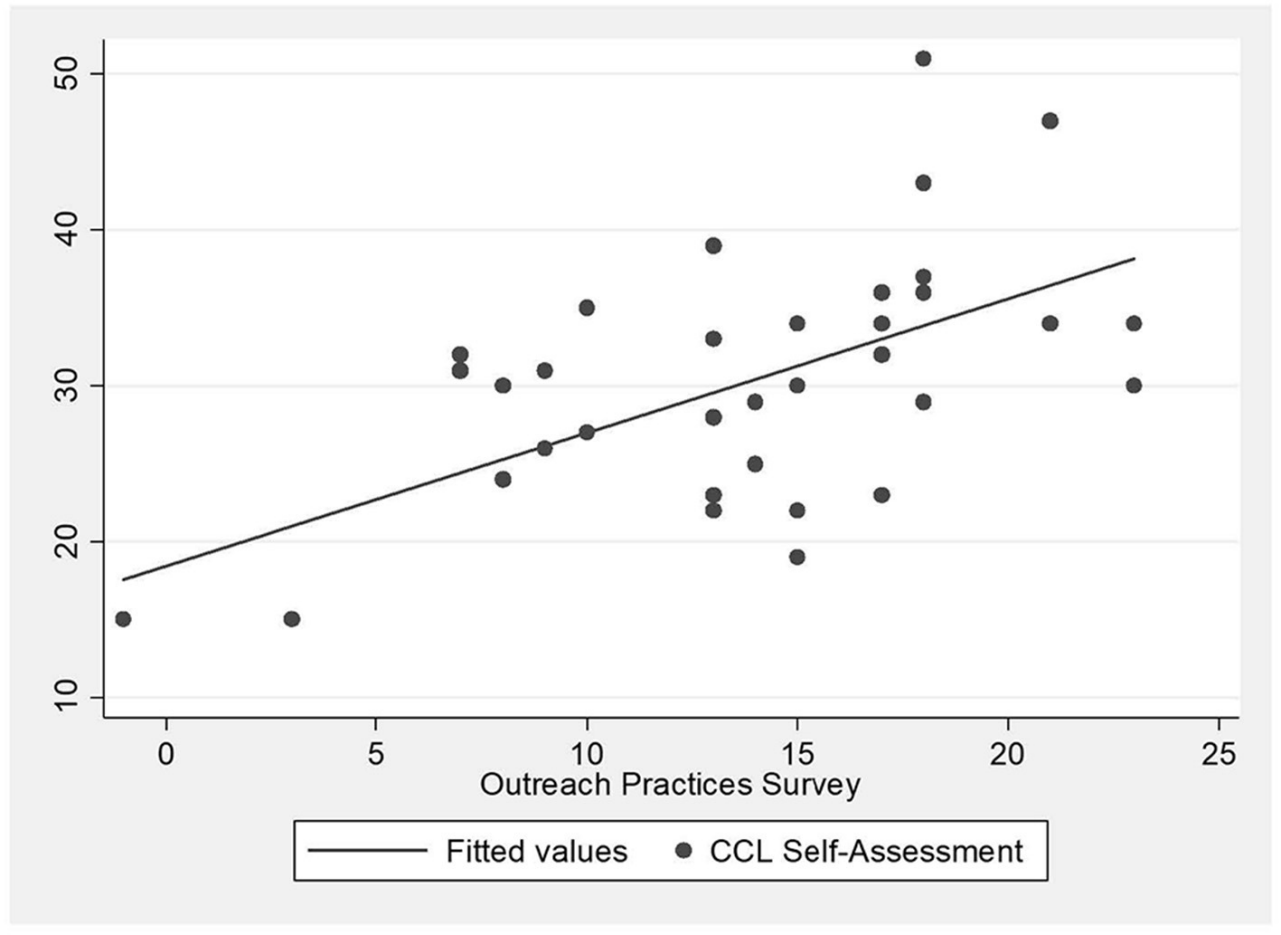
Comparison of scores of clinical-community linkage self-assessment survey and outreach practices survey.

We found sites that scored high on the CCL Self-Assessment measure also scored high on the Outreach Practices Survey, and vice-versa. Our primary analysis of the summary scores found significant results using simple linear regression (β: 0.89, *p* < 0.001) and Pearson's correlation coefficient (0.71, *p* < 0.001) (Table 3). We also compared the scores on a scatter plot (Figure 2), which shows a roughly linear association.

In our secondary analysis, we found that scores for four of the five domains (nature of the relationship, communication, referral process, feedback loop) correlated with the total score from the Outreach Practices Survey (Table 3).

## Discussion

We developed and validated a 15-item CCL Self-Assessment that covered five essential CCL domains: nature of the relationship, communication, referral process, feedback, and timeliness. The instrument performed well in a feasibility test with a group of YMCA Association staff. We developed the instrument based on an existing framework with established content validity and demonstrated criterion validity when compared to results from a conceptually related but distinct survey that also captured empirical practices related to partnership strength.

Our comprehensive literature review and expert feedback yielded an initial product that was close to meeting the needs of community organization staff and ensured our instrument filled a gap in the available literature. Expert review and cognitive testing with the intended audience showed that the CCL Self-Assessment was a pragmatic, useful, and desired tool and demonstrated face and construct validity. Feasibility testing with a group of community organizations enrolled in a CCL development study showed that the instrument was easy to administer, and that organizations are willing and able to fill it out. Criterion testing provided evidence of validity for the full survey, and for four of the five domains. Although adding feedback from the linking partner is an important part of a holistic evaluation and should be the focus of future research, the linking partner assessment is beyond the scope of our current work.

### Instrument Development and Think-Aloud Interviews

Including multiple perspectives and types of evidence throughout our process allowed us to identify stakeholder needs with more certainty. Reviewing the literature underscored the scarcity of CCL resources and the need for evaluative instruments. Think-aloud interviews echoed feedback heard during our expert review: that the instrument is important and useful to stakeholders, on the condition it be brief and actionable. Although respondents pointed out the survey would be most useful to leadership, it may be beneficial for organizations to assess differing perspectives throughout their workforce, regardless of position within the organization. Previous literature suggests that a key to successful partnerships is consistent awareness, understanding, and cohesiveness (e.g., high commitment, engagement and buy-in) of the partnership by staff at all levels of the organization (1).

### Feasibility Testing

The range and distribution of domain scores (presented in Table 3), and the similarity of the standard deviation suggested that the survey captured both low and high performance, and that respondents did not exaggerate their responses to make their performance appear higher. Individual item scores predicted corresponding domain scores, suggesting that none of the items were contrary to the latent concept they represented. Among our respondents, capacity to enroll new program participants (as captured by the *timeliness* question) was their greatest strength, while establishing a standard referral process with their clinical partners needed the most improvement. This result is consistent with previous literature showing barriers to creating system-level infrastructures for referrals between clinical and non-clinical organizations (6, 81).

### Criterion Testing

The CCL Self-Assessment showed validity in criterion testing for the overall survey and for four of the five domains. Testing the validity of all CCL Self-Assessment domains enabled us to diagnose potential problems in the assessment. The single-item timeliness domain had weak, non-significant correlations (β: 0.06, *p* = 0.23; Pearson's 0.35, *p* = 0.13). This suggests that timeliness may not have been captured in an appropriate level of detail or context in the Outreach Practices Survey, where it appeared as a single item in a checklist of barriers.

### Identifying Next Steps

In this paper we described a step-by-step process for developing and validating a pragmatic, formative assessment, and this approach is likely applicable to a range of settings where a formative self-assessment tool is thought to be needed. The initial results of the CCL Self-Assessment are promising and meet a pressing need: respondents want to better understand and strengthen their relationships with clinical partners. Next steps include testing the CCL Self-Assessment in the field to understand how people use it, and determining if and when it contributes to stronger CCLs. An important future consideration is to further develop the tool to include the perspective of the clinical partner. This would involve testing the same survey design with CCL dyads. If the tool demonstrates utility in improving CCLs, researchers should identify how to disseminate it to community organizations and demonstrate how to use it, which will likely include working with community organization leadership. In order to maintain our commitment to provide stakeholders with an actionable instrument, an important addition will be developing and testing an accompanying toolkit that maps directly to the self-assessment and provides step-by-step guidance for strengthening partnerships.

## Limitations

A danger with any formative scale is that it misses critical content and therefore has poor content validity. It is impossible to rule out the prospect that we failed to identify and include critical components of CCLs in our assessment. We attempted to limit this risk by initially basing this work on the CCRM Framework and a review of the related literature, followed by iteratively revising the assessment through a series of think-aloud interviews with our intended audience (community organization staff). This limits, but does not eliminate, the danger that one or more important domains of CCLs were missed. Additionally, we attempted to mitigate the drawbacks of the think-aloud interviews. Our sample was ultimately restricted to the greater Seattle area of Washington State. However, we attempted to provide more generalizable insights into how useful the CCL Self-Assessment would be to a broader group of people and situations by identifying diverse stakeholder needs by engaging staff at different levels in organizations, and from multiple sectors of community based organizations (i.e., faith-based organizations, health promotion programs, senior centers, cancer support, non-governmental organizations, community health programs, and the department of health). To address the fact that think-aloud interviews are prone to subject resistance (73), we trained the interviewer to guide the informant and probe appropriately. Of note, we conducted feasibility testing and criterion validation in the context of an ongoing study, so participants could differ in important ways from similar staff at community organizations in non-research settings. However, we asked for a second respondent, who was not the primary contact in the study, and may more closely represent in-field participation. This practice aligned well with study findings showing the importance of gaining multiple perspectives in an organization (82). Lastly, the scope of our work only included the community organization, and would be improved by the inclusion of the clinical entity.

## Conclusion

We described the development of a self-assessment tool for community organizations to evaluate the strength and areas for improvement of their clinical partnerships. Our research showed that having an instrument to evaluate linkages is important (a key component to developing pragmatic measures) to stakeholders, if that instrument is brief, accessible, and pragmatic. We also demonstrated face, content, and construct validity of the CCL Self-Assessment survey and tested criterion validity with a conceptually related but distinct survey that captured empirical practices associated with partnership strength.

Initial results support the feasibility of the instrument, and suggest that the CCL Self-Assessment survey may be used by community organizations to identify the strengths and weaknesses of their linkages. Next steps may include additional statistical validation and testing to determine how the CCL Self-Assessment survey works in the field, and the inclusion of the clinical entity.

## Data Availability Statement

The original contributions presented in the study are included in the article/supplementary files, further inquiries can be directed to the corresponding author/s.

## Ethics Statement

The studies involving human participants were reviewed and approved by the University of Washington Institutional Review Board. Written informed consent for participation was not required for this study in accordance with the national legislation and the institutional requirements.

## Author Contributions

SF, JH, MP-P, and MK: study conception and design. SF: data collection and draft manuscript preparation. SF, JH, MP-P, MK, and CH: analysis and interpretation of results and manuscript revision. All authors reviewed the results and approved the final version of the manuscript.

## Funding

This work was supported by a Health Promotion and Disease Prevention Research Center, Cooperative Agreement Number U48DP005013 from the Centers for Disease Control and Prevention, as well as grant support for REDCap electronic data-capture tools (UL1 TR002319, KL2 TR002317, and TL1 TR002318 from NCATS/NIH).

## Conflict of Interest

The authors declare that the research was conducted in the absence of any commercial or financial relationships that could be construed as a potential conflict of interest.

## Publisher's Note

All claims expressed in this article are solely those of the authors and do not necessarily represent those of their affiliated organizations, or those of the publisher, the editors and the reviewers. Any product that may be evaluated in this article, or claim that may be made by its manufacturer, is not guaranteed or endorsed by the publisher.

## Footnotes

^1^The agency also created several resources to guide research and evaluation of effective clinical-community resource relationships (56).
